# From a Few Cardiovascular Risk Factors to the Prediction of Age at Death: The Shifting Interests of Cardiovascular Epidemiologists

**DOI:** 10.3390/jcdd12020035

**Published:** 2025-01-21

**Authors:** Alessandro Menotti, Paolo Emilio Puddu

**Affiliations:** 1 Association for Cardiac Research, Via Voghera, 31, 00182 Rome, Italy; amenotti2@gmail.com; 2 EA 4650, Signalisation, Électrophysiologie et Imagerie des Lésions D’ischémie Reperfusion Myocardique, Normandie Université, Esplanade de la Paix, 14000 Caen, France

**Keywords:** cardiovascular risk factors and diseases, cancer, all-cause mortality, age-at-death

## Abstract

We describe the changing research interests and goals of the responsible investigators of the Italian Rural Areas (IRA) of the Seven Countries Study of cardiovascular diseases (CVD) during a period of 60 years, dealing with a cohort of middle-aged men. Our initial interest was to discover the basic risk factors of coronary heart disease (CHD). Subsequently, the same problem was tackled regarding stroke and heart diseases of uncertain etiology. Later on, cancer deaths also became an end-point for which risk factors were investigated. The long duration of the study and the fact that CVD and cancer fatalities already cover 70% of all-cause mortality prompted the idea to focus on all-cause mortality, and particularly on age-at-death when the follow-up period reached 61 years together with the extinction of the cohort. At that point, a larger number of risk factors measured at baseline, including those which were unable to predict CVD, became the determinants of all-cause mortality and age-at-death, a metric that summarizes the life-span of health and disease. This study is supported by the presentation of data derived from published papers.

## 1. Foreword

This is an attempt to reproduce the changing approaches of a group of cardiovascular epidemiologists in their attitude towards research goals, beginning in the early 1960s when field epidemiology of cardiovascular diseases began. The purpose is to describe, as an example, our personal experience within the Italian Research Group of the Seven Countries Study of cardiovascular disease (SCS). It is shown that the research targets and interests did change along 60 years of work, starting with the enrollment of a cohort of middle-aged men in the search of risk factors for coronary heart disease (CHD) and subsequently with the opportunity to follow-up this cohort for more than 60 years until its extinction [1].

This is not a new analysis, but only a presentation and some comments of selected data derived from the published quoted papers herein.

## 2. The Italian Rural Areas of the SCS

We refer to the Italian Rural Areas (IRA) of the SCS from 1960, when a baseline examination was made on 1712 middle-aged men (aged 40–59) out of 1735 listed in a roster of two rural municipalities, with an entry participation rate of 98.7% [1].

The entry examination included the collection of family and social data; lifestyle behaviors including smoking, motion, and dietary habits; a series of anthropometric measurements; a few biochemical and biophysical measurements; diagnoses of majorly prevalent diseases obtained using a complex medical examination; and the recording of a resting and post-exercise electrocardiogram and resting spirometry measurements [1]. All measurement techniques underwent strict standardization procedures and several variables were excluded, including those not suitable for standardization. These included blood glucose, HDL cholesterol, and triglycerides levels; some coagulation indicators; chest X-rays; and others. Follow-up included quinquennial field re-examinations (up to year 40 of follow-up), complex procedures to find additional data on the incidence of major cardiovascular diseases, and the collection and coding of mortality data up to year 61 of follow-up when the cohort was practically extinct (among the 1712 men examined at entry, there were 1708 deaths, three survivors and one lost to follow-up after 50 years). This was a great achievement of the study, and is probably unique in the history of field epidemiology.

## 3. Coronary Heart Disease (CHD) and Its Risk Factors

The first approach was to replicate the analysis made by prototypal studies in this field, such as the Minnesota Professional and Business Men study [2] and the Framingham Heart Study [3], both in the USA. This analysis consisted of a few variables, initially called “factors of risk” and later on “risk factors”, that were shown to predict the occurrence of new CHD events, either fatal or non-fatal, during the first few years of follow-up. These included age, blood pressure, serum cholesterol, and smoking habits, and, in our study, they were predictive as expected [1,4,5], confirming their universal role.

The availability of many other potential risk factors prompted the idea to test the role of many other variables that were measured at baseline. However, the outcome of this approach was somewhat disappointing since the predictive role of most of the above potential risk factors was present only in some models and not in others, and at some length of follow-up and not in others. This was likely due to their lack of influence, their inter-correlation with other basic risk factors, the saturation effect of multivariate models, or even multi-collinearity problems. In the end, beyond confirming the role of the four classic risk factors, only the behavioral habits of the practice of vigorous physical activity and following a diet of Mediterranean style showed a large protective role from CHD fatalities until the end of the 61 years of follow-up and could be added to the four classical risk factors [1,4,5], while the role of the other variables was almost irrelevant and inconsistent along time. Moreover, within dietary habits, a dietary intake of saturated fat directly predicted CHD events, marginally for stroke yet irrelevant for Heart Disease of Uncertain Etiology (HDUE) events [6].

## 4. Risk Factors for Other Major Cardiovascular Diseases

Only after some decades, interest moved to other forms of CVD, in particular strokes and large groups of heart diseases manifested only with heart failure, arrhythmia, and blocks in the absence of a clear etiology, like typical coronary syndromes (myocardial infarction, acute ischemic attacks, and sudden coronary deaths). We named these: Heart Diseases of Uncertain Etiology (HDUE) [1]. In the case of stroke, some difficulties arose from the smaller numbers compared with CDH events and by the impossibility of segregating hemorrhagic from thrombotic strokes, except in a small proportion.

The analytical approach for stroke and HDUE was the same as for CHD, and in the end, different outcomes were found for those two end-points. The main issue was that serum cholesterol was not predictive of or even inversely related to HDUE but was marginally predictive for stroke (only after 61 years of follow-up), while blood pressure and smoking habits were directly related risk factors for both conditions [1]. The Mediterranean diet did not provide protection, while physical activity had an uncertain role in regards to stroke and HDUE. Since the follow-up period was very long, with the cohort close to extinction, the evaluation of age-at-death was possible and showed lower levels for CHD, higher levels for HDUE, and intermediate levels for stroke, contributing to the differentiation of the characteristics in the natural history of those three major types of CVD [1,5]. Also, the first occurrence of disease for CHD, HDUE, and stroke had the same temporal shape.

## 5. Prediction of Cancer

This step was substantially delayed due to the fact that within the many risk factors tested for CVD, almost none, except smoking habits, could be reasonably expected to play a specific role in the prediction of cancer deaths.

An analysis produced after 15 years of follow-up and another one after 61 years (with the extinction of the cohort) confirmed that smoking habits and diabetes were strong and positive predictors of cancer, while the protective role of the Mediterranean Diet became evident only after 61 years of follow-up [5]. Moreover, after 61 years of follow-up, cancer deaths were associated with corneal arcus and xanthelasma. The last two risk factors were, incredibly enough, not predictive of major CVD deaths, although they were indicators of abnormal lipid metabolism. Details of the locations of single types of cancer did not provide valuable indications (except lung cancer as related to smoking habits) due to the relatively small numbers involved.

## 6. All-Cause Mortality, Extinction of the Cohort, and Age-at-Death

In the early phases of the study, predictive models for all-cause mortality at different horizons of follow-up were not omitted but were somewhat disregarded due to the fact that the prediction was limited by the use of CVD risk factors. However, our interest increased considering that CVD and cancer, as tested so far, were the causes of death of more than 70% of all-cause mortality. This prompted the idea to produce many multivariate models including all possible risk factors and personal characteristics measured at baseline examination in the 1960s.

There were two end-points, i.e., all-cause mortality and age-at-death. All-cause mortality became the dependent variable of the Cox proportional hazards models. Although practically all men had died, the models produced reliable findings because they included the time variable. Age-at-death, instead, became the dependent variable of the linear regression models [7].

Age-at-death is an old demographic metric that was recently re-evaluated [8,9,10,11,12]. It roughly corresponds to the expectancy of life but can only be properly used if the study population is extinct or almost extinct. At the end of the follow-up period, 20 personal characteristics measured at baseline provided significant associations with the length of life expressed by age-at-death.

## 7. Interpretation of Tables

Table 1 summarizes the relationships of risk factors available in the study with a few major fatal end-points described above and ordered according to the time-changing interest for those end-points, related to Cox models.

The indications of direct (significant direct association) and inverse (significant inverse association) relate to cases where the predicting power was constantly present for both short and long follow-up periods, ending with the extinction of the cohort. In the case of no indications, the association of the risk factor with the outcome was absent or was present in only some occasions, characterized by a peculiar follow-up duration, or was associated with the presence of very few other covariates or only carrying marginal significance. One of the problems was that a potential but weak risk factor may not reach significant association with the events due to the saturation effect, strong correlations with other factors, or even multi-collinearity effects. However, several of these risk factors became definitely predictive when forced into models containing all-cause deaths or age-at-death as end-points and with a long follow-up period of observation.

In the area of all-cause death and age-at-death, as well in the other areas, some available risk factors were deliberately disregarded to avoid collinearity problems. This was the case at least for diastolic blood pressure to avoid problems with systolic blood pressure, forced expiratory volume in ¾ sec to avoid problems with vital capacity, and urine glucose to avoid problems with the clinical diagnosis of diabetes.

Models with age-at-death as the end-point used the same risk factors tested in the Cox models for all-cause mortality and were solved with multiple linear regression (MLR). Although expressed in a different way, the same risk factors were predictive in both types of models. However, an advantage of the latter dealing with age-at-death as the end-point was the possibility to compute the number of years gained or lost as a function of each risk factor and to produce theoretical estimates of lifelong duration as a function of arbitrary combinations of risk factors and their levels.

Risk factors significantly predicting all-cause mortality and age-at-death are summarized in Table 2 where each risk factor, either discrete-binomial or represented by an amount roughly corresponding to one standard deviation if it is continuous in nature, is associated with a number of years gained or lost depending on the algebraic direction of their association with the end-point, as derived from MLR predicting age-at-death.

There are several groups of characteristics that significantly predict age-at-death, moving from social–familiar data to anthropometric measurements, lifestyle behaviors, classical cardiovascular risk factors, and clinical diagnoses and characteristics. Apart from being free from major diseases and other clinical signs, the most important characteristics associated with a high age-at-death were never having smoked, undertaking vigorous physical activity, following the Mediterranean Diet, and having low blood pressure and high vital capacity levels. A special case was that of body mass index, which was significant for all-cause mortality and age-at-death but only if treated in a parabolic way, with favorable outcomes for intermediate levels and bad outcomes for extreme upper and lower levels.

In the MLR predicting age-at-death, each risk factor was automatically adjusted for all the others, with the consequence that the levels of the multivariate coefficients are somewhat smaller if compared with an analysis run using the factors one-by-one, with a loss, in the levels of coefficients, of 20 to 100%. This effect is documented in the right-end column of Table 2, where the years gained or lost were recomputed without the adjustment of other variables. In a few cases (i.e., age and subscapular skinfold), there were also unexplained differences in the algebraic sign when comparing the two approaches. The above problems were partly compensated for by the fact that the years gained or lost can, when adjusted for other variables, be algebraically added for estimates of age-at-death, suggesting that piling up several of them together may result in rather large differences in the estimated age-at-death.

## 8. Lessons from a 60-Year-Long Study

A few lessons were learned through simply reviewing the predictive power of the variables measured at baseline.

- CHD, stroke, and HDUE are different CVD types since they have only two risk factors in common (blood pressure and smoking habits), while serum cholesterol is specifically predictive of CHD, marginally predictive of stroke, and irrelevant for HDUE (or even inverse). Moreover, the age of occurrence is younger for CHD, intermediate for stroke, and definitely older for HDUE, and the same ranking applies to age-at-death [1,5]. The real etiology of HDUE is unknown and deserves further research, although it has attracted little attention from investigators.
- The same block of major risk factors typical for major CVD, and particularly CHD, do not predict cancer deaths as claimed by Cardio-Oncology scholars [5]. Only smoking habits and the protective role of the Mediterranean diet are common predictors of CHD and cancer events.
- Many of the hypothesized CVD risk factors were not consistently predictive of CVD but played a substantial role in the prediction of all-cause mortality and age-at-death, probably through different and largely undefined mechanisms.
- The 20 risk factors significantly associated with age-at-death are a subset of those hypothesized and measurable more than 60 years ago, and therefore, there is hope to add many more of them to cover the parts of this prediction still unrealized. In fact, several other risk factors were studied in more recent investigations, but they were not measurable or were even unknown in the early 1960s. However, a few studies measured a large number of possible determinants, but no other have likely reached the stage of cohort extinction.
- If observational epidemiology is a prelude to prevention, it becomes mandatory to take into serious consideration any type of possible determinant of all-cause death and age-at-death (including genetic traits), since intervention into these may theoretically prolong the expectancy of life, although the goal is to increase the number of healthy years in the overall years of life.

It is not completely clear whether the same path has been followed by other investigators. However, in our case the decision was to prolong the follow-up until the extinction of the cohort.

New studies are likely not needed, however efforts should be made to collect and put together data on risk factors for all-cause mortality, which surely exist in the majority of current population studies, to see whether new risk factors contribute to the prediction of how life might be lengthened. Of course, studies involved in this operation should have very long follow-up data on all-cause mortality and could possibly approximate the extinction of cohorts, therefore adopting age-at-death as their end-point.

## Figures and Tables

**Table 1:** A summary of the relationship of risk factors with various types of fatal events during a follow-up of 61 years and the extinction of the cohort [1,4,5,6,7].

Risk Factors	Predicting				
	CHD	HDUE	Stroke	Cancer	All-Cause
Age	Direct	Direct	direct	direct	direct
High socio-economic status	----	----	----	----	----
Father early death	----	----	----	----	----
Mother early death	----	----	----	----	direct
Marital status (married)	----	----	----	----	inverse
Sedentary physical activity	direct	----	----	----	direct
Moderate physical activity	inverse	----	----	----	inverse
Vigorous physical activity	inverse	----	----	----	inverse
Smoker	direct	direct	direct	direct	direct
Ex-smoker	inverse	----	----	----	inverse
Never smoker	inverse	----	----	----	inverse
Non-Mediterranean diet	direct	----	----	direct	direct
Intermediate diet	inverse	----	----	----	direct
Mediterranean diet	inverse	----	----	----	direct
Body mass index	if treated in a parabolic way: direct for extreme levels predicting all-cause mortality				
Subscapular skinfold	----	----	----	----	inverse
Trunk/height ratio	----	----	----	----	----
Shoulder/pelvis shape	direct	----	----	----	----
Laterality/linearity index	----	----	direct	----	direct
Arm circumference	----	----	----	----	inverse
Systolic blood pressure	----	----	----	----	direct
Heart rate	direct	----	----	----	direct
Vital capacity	inverse	----	inverse	----	inverse
Serum cholesterol	direct	----	direct	----	direct
Urine protein	----	----	----	----	----
Corneal arcus	----	----	----	direct	direct
Xanthelasma	----	----	----	direct	direct
Cardiovascular diseases	----	----	----	----	direct
Cancer	n.a.	n.a.	n.a.	----	direct
Diabetes	----	----	----	direct	direct
Chronic bronchitis	----	----	----	----	direct
Silent resting ECG abnormalities	----	----	----	----	----
Silent post-exercise ECG abnormalities	----	----	----	----	----

Direct = direct and significant association with events; inverse = inverse and significant association with events. Findings derived from Cox models predicting the various end-points [5,7]. n.a. = not available.

**Table 2:** A list of significant factors associated with age-at-death in 61 years, derived from a multiple linear regression model [8].

Risk Factor	*p* of Coefficient	Delta	Years Gained (+) or Lost (−)	Years Gained (+) or Lost (−) Without Adjustment for the Other Covariates
Age, years	<0.0001	+5	+1.11	−0.65
Mother early death, yes–no, 1–0	0.0024	+1	−1.96	−2.39
Marital status, yes–no, 1–0	0.0182	+1	+2.12	+2.51
Sedentary physical activity, yes–no, 1–0	Reference for Vigorous physical activity			
Vigorous physical activity, yes–no, 1–0	0.0133	+1	+2.39	+4.22
Smoker, yes–no, 1–0	Reference for Never smokers			
Never smoker, yes–no, 1–0	<0.0001	+1	+2.71	+3.50
Non-Mediterranean diet, yes–no, 1–0	Reference for intermediate and Mediterranean Diet			
Intermediate diet, yes–no, 1–0	0.0030	+1	+1.98	+2.23
Mediterranean diet, yes–no, 1–0	0.0004	+1	+2.71	+3.84
Body mass index, kg/^2^ Significant in parabolic shape: intermediate levels directly related to age-at-death				
Laterality/Linearity index, rate of diameters	0.0242	+2	−0.50	−0.26
Subscapular skinfold, mm	0.0002	+6	+1.70	−0.11
Arm circumference, mm	<0.0001	+25	+1.57	+1.43
Systolic blood pressure, mmHg	<0.0001	+20	−2.09	−2.69
Vital capacity, L/m^2^	<0.0001	+0.25	+1.66	+2.13
Serum cholesterol, mg/dL	0.0004	+40	−0.95	−1.14
Corneal arcus, yes–no, 1–0	0.0197	+1	−1.90	−3.77
Xantelasma, yes–no, 1–0	0.0293	+1	−4.66	−8.03
Cardiovascular diseases, yes–no, 1–0	0.0153	+1	−3.10	−6.03
Cancer, yes–no, 1–0	<0.0001	+1	−20.10	−18.35
Diabetes, yes–no, 1–0	0.0454	+1	−2.48	−4.21
Chronic bronchitis, yes–no, 1–0	0.0047	+1	−3.05	−4.16

Delta = unit of measurements used for the estimate of years gained or lost, either multivariately or univariately.
